# A Quasi-Steady Lifting Line Theory for Insect-Like Hovering Flight

**DOI:** 10.1371/journal.pone.0134972

**Published:** 2015-08-07

**Authors:** Mostafa R. A. Nabawy, William J. Crowthe

**Affiliations:** School of Mechanical, Aerospace and Civil Engineering, The University of Manchester, Manchester, United Kingdom; Brown University, UNITED STATES

## Abstract

A novel lifting line formulation is presented for the quasi-steady aerodynamic evaluation of insect-like wings in hovering flight. The approach allows accurate estimation of aerodynamic forces from geometry and kinematic information alone and provides for the first time quantitative information on the relative contribution of induced and profile drag associated with lift production for insect-like wings in hover. The main adaptation to the existing lifting line theory is the use of an equivalent angle of attack, which enables capture of the steady non-linear aerodynamics at high angles of attack. A simple methodology to include non-ideal induced effects due to wake periodicity and effective actuator disc area within the lifting line theory is included in the model. Low Reynolds number effects as well as the edge velocity correction required to account for different wing planform shapes are incorporated through appropriate modification of the wing section lift curve slope. The model has been successfully validated against measurements from revolving wing experiments and high order computational fluid dynamics simulations. Model predicted mean lift to weight ratio results have an average error of 4% compared to values from computational fluid dynamics for eight different insect cases. Application of an unmodified linear lifting line approach leads on average to a 60% overestimation in the mean lift force required for weight support, with most of the discrepancy due to use of linear aerodynamics. It is shown that on average for the eight insects considered, the induced drag contributes 22% of the total drag based on the mean cycle values and 29% of the total drag based on the mid half-stroke values.

## Introduction

The classical lifting line theory (LLT), developed by Prandtl a century ago provided the first satisfactory analytical treatment for the evaluation of the aerodynamics of a finite wing [1–6]. The LLT laid the foundation for understanding the aerodynamics of flight, and is still widely used today to provide accurate predictions of the lift and induced drag for 3d wings [6]. The solutions delivered by the LLT are closed form and they are many orders of magnitude faster to evaluate compared to higher order computational methods; they are also able to provide deep insight into how different wing parameters affect the aerodynamic performance [6].

The physical foundation of the LLT is based on Prandtl's hypothesis that the lift of a finite wing is reduced compared to the lift of an infinite wing due to the change of the local flow direction induced by the free vortices in the wake. The Kutta-Joukowski theorem can then be applied at each wing section, which is assumed to behave as a 2d wing at a modified angle of attack referred to as the *effective angle of attack*. This concept led Prandtl to his well-known linear equation governing the circulation on a finite lifting surface, which will be formally introduced later in this work in section ‘LLT fundamental equations’. Because the obtained governing equation is of an integro-differential type, there exists no unique mathematical procedure to solve it, and throughout the past century different mathematical methods have been proposed to handle the problem [7]. The most well-known solution methodology is that presented by Glauert [8] who provided a solution in the form of an infinite Fourier sine series with the series coefficients obtained from the collocation method.

Whilst the LLT is usually used for the aerodynamic modelling of high aspect ratio, planar, fixed wings in steady flows, the long reach of Prandtl's insight is demonstrated through the various adaptations presented over the years that have enabled much broader applicability of his original model [9]. With few adaptations, the LLT has been successfully used to predict the aerodynamics of a wide variety of lifting surfaces under a wide variety of flow conditions. Jones [10] proposed a simple correction for the LLT which he showed could bring the lifting line result into close agreement with the lifting surface result over an extended range of wing aspect ratio, hence improving accuracy of the LLT for low aspect ratio wings. Phillips and Snyder [9] extended the lifting line formulation so that it can be used for non-planar wings with arbitrary camber, sweep and dihedral. Sclavounos [11] developed an unsteady lifting line treatment for wings of large aspect ratio undergoing time-harmonic oscillations where he showed in the zero-frequency limit that it reduces to the Prandtl's lifting line theory, whilst for high frequencies it tends to the two-dimensional strip theory. Mehrle [12] extended Multhopp's quadrature method to the calculation of the circulation of cyclic periodic lifting systems, e.g. for wings operating in swirling flow. Anderson *et al*. [13,14] proposed a numerical iterative lifting line treatment that uses look-up tables of the sectional lift as a function of effective angle of attack for the use within flight conditions such as spins and high angles of attack manoeuvres.

The LLT has also been adopted for the evaluation of the aerodynamics of wings prescribing rotary and flapping motions. Conlisk [15] discussed the implementation of the LLT for rotary wings in hover, and highlighted the importance of accounting for the effect of the linear velocity variation along the blade on the bound circulation distribution. Leishman (see chapter 14 in [16]) provided a generic formulation of the LLT for rotary wing motions; whereas, Johnson (see chapter 10 in [17]) discussed the importance of adopting corrections to the LLT to handle specific rotary wing aerodynamic phenomenon such as wake periodicity.

Lifting line formulations very similar to that of fixed wings have been used in [18,19] for the mathematical modelling of the avian flight power curve. Philips *et al*. [20] presented a LLT for forward flapping flight in which some unsteady flow effects were accounted for through the use of a 3d model of the vortex wake to evaluate the unsteadiness to a first order. For a review of lifting line models for flapping wings in forward flight, the reader is referred to [21].

For hovering flapping flight, two significant contributions have been presented. The first is by Sane [22] who proposed a semi-empirical lifting line blade model for hovering insects to investigate the mean induced flow over their bodies. However, the model relies on experimental data; hence, measurements are still required as inputs to the calculation. The second contribution is by Ansari *et al*. [23] who reviewed the use of lifting line blade theory based on the Glauert solution [8] in the context of insect-like flapping wings: a general description of the model was provided and some results for the variation of the mean lift with flapping frequency and wing shape were presented. However, their model relies on a linear aerodynamic representation which would significantly over-estimate the lift and induced drag at high angles of attack where insects are known to operate. Additionally, other relevant aerodynamic phenomena such as wake periodicity are not included.

Within the last two decades, there has been an increased interest in studying insect flight, particularly in hover. Most of the studies in this field have been either experimental or numerical with relatively few analytical contributions. The most widely used analytical class of models for the prediction of hovering insects aerodynamics are the so-called quasi-steady models such as those developed in [24–27]. These models assume equivalence of the instantaneous aerodynamic forces on a flapping wing with the forces generated on the same wing moving steadily at the same instantaneous velocity and angle of attack [28]. However, most of the available models as in [24–26] relied on experimental data to define the flapping translational force coefficients which are the primary contributor to the generated forces. Thus the applicability of such models is limited to a few test cases for which experimental data are available [29].

The aim of the present work is to provide a convenient theoretical treatment for evaluating the aerodynamics of insect-like wings in the translational phase of the flapping cycle. This work builds on the foundation laid by the authors in their previous contributions [29,30] which establishes a compact transparent treatment for the quasi-steady aerodynamics of hovering. The contribution of the present work lies in the novel reformulation of the LLT for application to estimating the translational forces for hovering wings and the subsequent insight that this brings to the flow physics. In particular, the modelling approach allows unique insight into the relative contribution of induced and profile drag for flapping wings; something that is currently missing in the available literature. Whilst the present contribution only considers quasi-steady effects, there is a logical path to include rotational and added mass effects as model extensions in the future that would enable capturing aerodynamic time history effects.

## Method

### Basic assumptions

The lifting line theory assumes a fluid that is incompressible and inviscid. Compressibility effects are negligible for application areas of interest. With regard to viscous effects, recent experimental measurements [31] have demonstrated that insect-like flapping wing aerodynamics depends weakly on Reynolds number, and numerical studies [32] demonstrated that the flows are well modelled by the inviscid Euler equations. Nevertheless, and following the general practice within the LLT, the Reynolds number effect is taken into account in the two dimensional properties of the wing section represented through the section lift curve slope.

The wing is assumed to be an infinitesimally thin and un-cambered rigid flat plate with zero spanwise twist and zero sweep. Wing twist about a spanwise axis can be included as an alteration to the wing geometric angle of attack.

The lifting line theory is valid as long as the Kutta condition is satisfied, and in general this will be the case if there is an absence of classical wing stall [6]. For the current problem, the formation of a leading-edge vortex (LEV) on the wing top surface prevents classical wing stall [33,34,29]. The LEV is stable in the sense that it does not shed as the wing motion progresses, and allows the flow over the upper surface of the wing to separate at the leading edge but subsequently reattach upstream of the trailing edge [34]. The Kutta condition is therefore established at the trailing edge at angles of attack beyond which classical stall would occur for wings where no LEV is present [34,33,29].

Other secondary aerodynamic effects from wing pronation and supination as well as the wing-wing interactions (clap-and-fling) are not included in the current model. Thus the current modelling treatment is consistent with the well known ‘revolving wing’ concept [31,35–39] which captures the quasi-steady aerodynamics between stroke reversals.

### LLT fundamental equations

The wing is modelled as a bound vortex of strength Γ(*r*) at the aerodynamic centre. The goal is to determine Γ(*r*) as a function of the wing geometric properties. The Kutta-Joukowski theorem is used to obtain the lift per unit span [8,16]:

$$dL(r)=\rho V(r)\Gamma (r)dr=\frac{1}{2}\rho {\left(V(r)\right)}^{2}c(r)drC_{l\alpha ,2d}\left(\alpha_{g}-\alpha_{i}(r)\right),$$ (1)

where *ρ* is the air density, *V*(*r*) is the sectional flow speed along the wing length, *r* is wing radial position measured from the wing root, *c* is the chord, *C*_lα,2d_ is the 2d-aerofoil lift curve slope, *α*_*g*_ is the wing geometric angle of attack and *α*_*i*_ is the induced angle of attack. Thus Γ(*r*)is obtained as:

$$\Gamma (r)=\frac{1}{2}c(r)C_{l\alpha ,2d}\left(V(r)\alpha_{g}-w(r)\right),$$ (2)

where *w*(*r*) is the induced downwash velocity distribution along the wing length determined by [4,8]:

$$w(\overset{⌣}{r})=\frac{1}{4\pi }\int_{-R}^{R}\frac{d\Gamma }{dr}\frac{dr}{\overset{⌣}{r}-r},$$ (3)

where $\overset{⌣}{r}$ is the wing station at which the downwash is calculated, and *r* is the location of vortices responsible for the downwash.

Eq 2 represents Prandtl's fundamental lifting line equation. In order to apply it to insect-like wings in hover, we rewrite it as:

$$\Gamma (r)=\frac{1}{2}c(r)C_{l\alpha ,2d,ef}\left(V(r)\alpha_{eq}-w_{ef}(r)\right).$$ (4)

The above equation represents the basis for the developed lifting line theory for hovering wings which, in the present work, will be referred to as LLT_hw_. Three main adaptations are introduced in Eq 4. These correct for (1) non-linear aerodynamics of the lift curve, (2) non-ideal induced downwash effects, and (3) planform effects on the 2d lift curve slope. Each of these adaptations is now considered in detail.

### Adapting the LLT for non-linear aerodynamics

The primary adaptation we make to the classical LLT is the introduction of the concept *equivalent angle of attack* to account for non-linearity in the wing lift curve. This *equivalent angle*, *α*_*eq*_, is defined as *the geometric angle of attack within the linear aerodynamic representation that will provide the same lift coefficient of the 3d wing within a non-linear aerodynamic representation*. The original LLT formulation assumes a linear lift curve for the wing; i.e. the 3d wing lift coefficient, *C*_*L*_, is proportional to the geometric angle of attack:

$$C_{L}\propto \alpha_{g}.$$ (5)

However, for an insect-like wing in hover, the lift coefficient increases to a maximum at a geometric angle of attack of 45 degrees and then decreases back to zero at a 90 degrees angle of attack. Previous studies [25,26,29,40,41] have shown that this behaviour can be adequately represented by the trigonometric relationship:

$$C_{L}\propto \text{sin}\alpha_{g}\text{cos}\alpha_{g}.$$ (6)

Experiments on revolving and flapping wings [31,35–38,40] show that despite its simplicity the function sin *α*_*g*_ cos *α*_*g*_ provides an excellent representation of the variation of the measured steady lift coefficient with geometric angle of attack. The physical foundation of the sin *α*_*g*_ cos *α*_*g*_ variation is based on the assumption that pressure forces dominate over skin friction forces for this type of flow, and the magnitude of the normal force coefficient is proportional to sin *α*_*g*_; for more details on the physical foundations of Eq 6, the reader is referred to references [40,29,35]. By comparison of Eqs 5 and 6, we derive an equivalent angle of attack expression as:

$$\alpha_{eq}=C_{\alpha_{g}}\alpha_{g},$$ (7)

where

$$C_{\alpha_{g}}=\text{sin}\alpha_{g}\text{cos}\alpha_{g}/\alpha_{g}.$$ (8)

**Fig 1:**
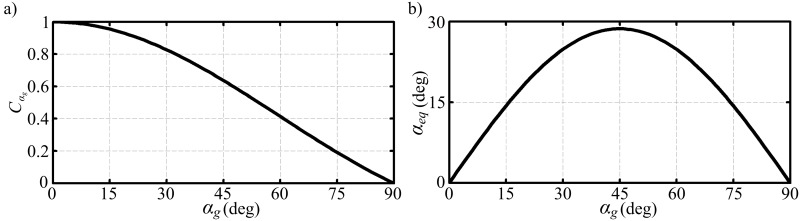
The equivalent angle of attack concept. Variation of (a) the correction term, and (b) the equivalent angle attack against the geometric angle of attack.

Fig 1 shows the correction term, $C_{\alpha_{g}}$, and the equivalent angle of attack, *α*_*eq*_, variations against the geometric angle attack. The maximum lift coefficient at a 45 degrees geometric angle of attack is achieved with a 29 degrees equivalent geometric angle of attack within the linear aerodynamics representation. At small angles of attack (*α*_*g*_ ≤ 15°), the equivalent angle of attack is almost equal to the geometric angle of attack meaning that the LLT_hw_ converges to the original LLT at low angles of attack. On the other hand, at very high angles of attack (*α*_*g*_ → 90°) the equivalent angle of attack reduces back towards zero as required by basic geometric considerations.

By applying the above adaptation within the LLT expressions, the quasi-steady non-linear lift curve behaviour essential to the insect-like flapping wing problem is well captured. An important aspect of this proposed technique is that no alterations to the fundamental LLT equations are required, and the underlying physics of the LLT is well preserved. Whilst the concept of the equivalent angle of attack is quite simple and appears as an obvious approach to handle the problem, it has to our knowledge not been attempted before either within the context of hovering insect-like wing problem or within any other non-linear aerodynamic treatment of a lifting surface. The equivalent angle of attack approach has some similarity with other techniques for implementing nonlinear aerodynamics for post stall applications such as ‘decambering approach’, for example described in [42], however these approaches typically require an iterative solution procedure. The present method does not require iteration because there is a single continuous function (Eq 6) that defines the overall lift coefficient variation of the wing as a function of angle of attack up to 90 degrees that can be easily inverted. This allows the original nonlinear problem to be transformed into an equivalent linear LLT problem. For more general cases with complex post stall aerodynamics and more arbitrary lifting surface arrangement no such convenience is available and it is necessary to iterate a solution.

The proposed adaptation has some similarities with the well known Prandtl-Glauert compressibility transformation [14] which allows solution of compressible flow problems using incompressible-flow calculation methods. The proposed LLT transformation allows solution of non-linear aerodynamic problems using linear aerodynamic calculation methods by applying linear aerodynamic methodologies to non-linear aerodynamic cases. We believe the proposed technique also opens the door for solution of other 3d lifting surface problems with non-linear aerodynamic behaviour.

### Adapting the LLT for non-ideal induced downwash effects

The second adaptation applied here is to account for non-ideal but physical effects that influence the downwash magnitude of the wing, including wake periodicity and effective flapping disk area. These effects are absent for fixed wings but must be considered for flapping wings [16,30,43,44]. To provide a simple modelling procedure for these effects, consider the actuator disk theory expression for the induced velocity magnitude in hover:

$$w=k_{ind}\sqrt{\frac{L}{2\rho S_{d,ef}}},$$ (9)

where *k*_*ind*_ is the well known *k*-factor to account for the non-uniformity in the downwash and is already accounted for in the lifting line formulation. However, there are other effects associated with flapping flight which are directly related to the downwash and need to be included in the lifting line formulation. These effects can be best explained through their effect on the effective disk area, *S*_*d*,*ef*_. For flapping flight, the disk area, *S*_*d*,*flap*_, is defined as [44]:

$$S_{d,flap}=2\phi R^{2},$$ (10)

where *ϕ* is the amplitude of the flapping stroke angle and *R* is the wing length from root to tip; however, to obtain an expression for *S*_*d*,*ef*_, a further modification is required as:

$$S_{d,ef}=2\phi R_{ef}^{2}.$$ (11)

The correction of *R* to *R*_*ef*_ accounts for the aerodynamic phenomena of wing tip losses due to discreteness and periodicity in the wake [16,17,44,45]. By quantifying the flow structure around a hovering model fruit fly wing using digital particle image velocimetry, Birch *et al*. [46] showed that experimental circulation falls to zero at around 85% of *R*. Sane [22] discussed this loss of lift near the tip and attributed it to tip losses due to wake periodicity.

Now, by simple factorisation [16], the downwash expression (Eq 9) can be written as:

$$w=k_{ind}\sqrt{\frac{L}{2\rho S_{d,ef}}}=k_{ind}\sqrt{\frac{L}{2\rho \pi R^{2}\left(2\phi /\pi \right)B^{2}}}=k_{ind}k_{per}k_{flap}\sqrt{\frac{L}{2\rho \pi R^{2}}},$$ (12)

where,

$$k_{per}=\frac{1}{B}=\frac{R}{R_{ef}},$$ (13)

$$k_{flap}=\sqrt{\frac{\pi }{2\phi }}.$$ (14)

Therefore, the overall downwash magnitude is increased due to the additional factor *k*_*per*_ *k*_*flap*_ compared to the case with no assumed wake periodicity effects and with the wing sweeping the whole circular disk area, i.e. *R* = *R*_*ef*_ and 2*ϕ* = *π*. Thus, from this simple momentum theory analysis, it can be seen that in the presence of these additional non-ideal effects a flapping wing has an overall induced velocity increased by the factor *k*_*per*_ *k*_*flap*_. We now develop the effective downwash definition in Eq 4 to account for these effects:

$$w_{ef}(\overset{⌣}{r})=\frac{k_{per}k_{flap}}{4\pi }\int_{-R}^{R}\frac{d\Gamma }{dr}\frac{dr}{\overset{⌣}{r}-r}.$$ (15)

In a previous contribution by the authors [30], numerical evaluations of the *k*_*per*_ and the *k*_*flap*_ parameters were presented for eight insect species. It was found that the value of *k*_*per*_ is clustered around 1.1; therefore without losing generality, this value will be used throughout this study. On the other hand, the value of *k*_*flap*_ varies considerably between different insects according to their flapping angle amplitude and thus insect specific values must be used.

### Correcting the 2d aerofoil lift curve slope

The final amendment to the LLT presented here is based on a well known correction to the 2d aerofoil lift curve slope originally proposed by Jones and usually referred to as the Jones edge-velocity correction [10,47,48]. Jones incorporated his correction into the 2d aerofoil lift curve slope leading to the concept of the effective 2d lift curve slope [48]:

$$C_{l\alpha ,2d,ef}=\frac{C_{l\alpha ,2d}}{E},$$ (16)

where *E* is the Jones correction evaluated as the ratio of the wing semi-perimeter to the wing length. Thus, the effective lift curve slope is a characteristic of the wing planform as well as the wing section [48]. This correction is most pronounced for wings with low aspect ratios, and as discussed in the introduction Jones showed that by applying his correction the LLT becomes more capable of capturing low aspect ratio effects.

**Fig 2:**
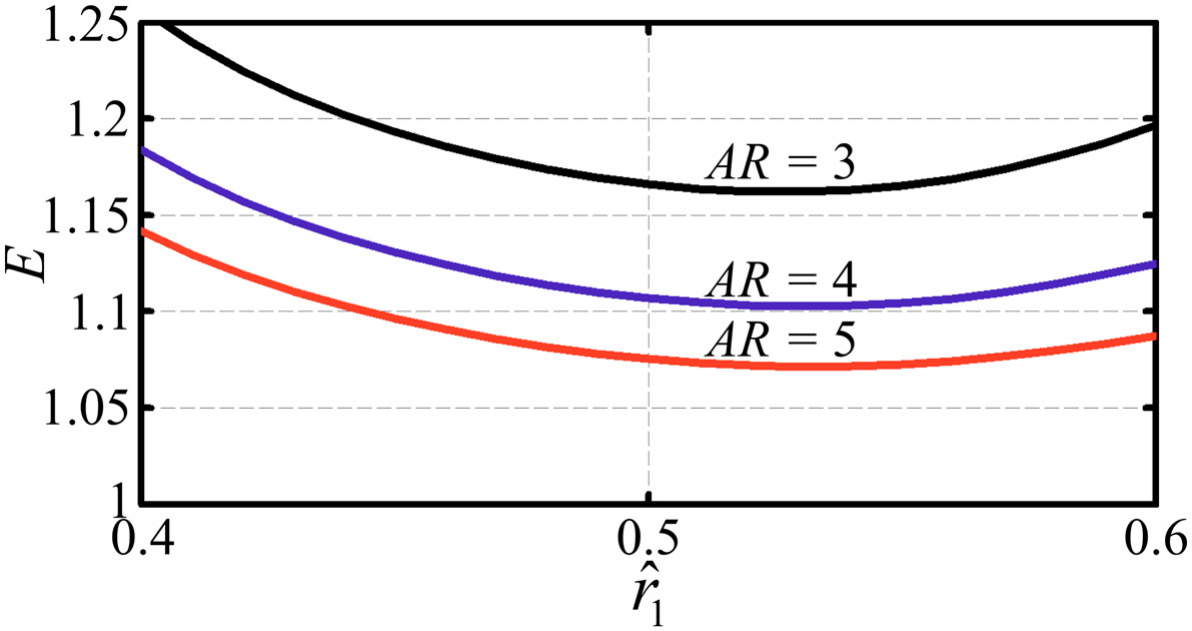
The edge correction. Variation of the Jones edge correction, *E*, for different combinations of wing aspect ratio and area centroid location. The wing planform is represented through the beta formulation (Eqs 17 and 18) for ${\hat{r}}_{1}$ values from 0.4 to 0.6 which is representative of the range found in nature. In this illustration, the wing is symmetric about the mid-chord.

Following Ellington [49], we define the wing chord distribution through a beta function representation, which provides a compact analytical description of the wing planform based on the wing length, the mean chord and the non-dimensional radial location of the wing centre of area:

$$c(r)=\bar{c}\left(\frac{{\hat{r}}^{p-1}{\left(1-\hat{r}\right)}^{q-1}}{\int_{0}^{1}{\hat{r}}^{p-1}{\left(1-\hat{r}\right)}^{q-1}d\hat{r}}\right)\;with\;\hat{r}=\frac{r}{R},$$ (17)

where the parameters are chosen as:

$$p={\hat{r}}_{1}\left(\frac{{\hat{r}}_{1}(1-{\hat{r}}_{1})}{{\hat{r}}_{2}^{2}-{\hat{r}}_{1}^{2}}-1\right),q=p\frac{\left(1-{\hat{r}}_{1}\right)}{{\hat{r}}_{1}},{\hat{r}}_{2}=0.929{\left({\hat{r}}_{1}\right)}^{0.732},$$ (18)

and ${\hat{r}}_{1}$ and ${\hat{r}}_{2}$ are the non-dimensional radii of first and second moments of area respectively. Insect wings typically have aspect ratios ranging from 2.5 to 6 [49,50], thus for the low aspect ratio cases the Jones correction is relevant. Fig 2 shows the variation of the Jones correction, *E*, for different combinations of the wing aspect ratio ($AR=R/\bar{c}$) and non-dimensional area centroid location (${\hat{r}}_{1}$).

The remaining unknown in Eq 16 is the 2d aerofoil lift curve slope, *C*_lα,2d_. For a flat plate at typical insect Reynolds numbers, experimental evidence suggests that *C*_lα,2d_ is slightly less than the theoretical value of 2*π* and takes a value of 0.09 deg^-1^ = 5.16 rad^-1^ [51–53]; thus this value will be used in this work.

### Solution methodology

Having introduced the essential adaptations to the LLT, we now solve Eq 4 using the well-known Glauert method [8,23]. First, the wing spanwise location is substituted with [8,47]:

r=−Rcosθ, (19)

where *θ* is a generic parameter used to define position along the wing. Given the symmetry of the problem, only one side of the wing is considered and thus *θ* varies from 0 to *π*/2. The circulation, Γ(*r*), is then expressed as a sine Fourier series as [8,47]:

$$\Gamma (r)=4RV(r)\sum_{m=1}^{\infty }a_{m}\text{sin}m\theta ,$$ (20)

where, for a hovering wing, the velocity along the wing length is given by the linear variation:

$$V(r)=\dot{\phi }r=-\dot{\phi }R\text{cos}\theta =-V_{tip}\text{cos}\theta .$$ (21)

Substituting Eq 20 into Eq 15 and performing integration using the Glauert integrals [54,23] leads to an expression for the effective downwash as a function of the radial position:

$$w_{ef}(r)=-k_{per}k_{flap}V_{tip}\sum_{m=1}^{\infty }\left(ma_{m}\frac{\text{cos}\theta \text{sin}m\theta }{\text{sin}\theta }+a_{m}\text{cos}m\theta \right).$$ (22)

The *α*_*m*_ coefficients in the above equation can be obtained using the well-known Glauert approach by equating Eqs 4 and 20 leading to:

$$\mu_{ef}\underset{\alpha_{eq}}{\underset{︸}{\text{sin}\alpha_{g}\text{cos}\alpha_{g}}}\text{sin}\theta \text{cos}\theta =\sum_{m=1}^{\infty }a_{m}\text{sin}\theta \text{cos}\theta \text{sin}m\theta +\mu_{ef}k_{per}k_{flap}\sum_{m=1}^{\infty }\left(a_{m}\text{sin}\theta \text{cos}m\theta +ma_{m}\text{cos}\theta \text{sin}m\theta \right),$$ (23)

where

$$\mu_{ef}=\frac{c(r)C_{l\alpha ,2d,ef}}{8R}.$$ (24)

In the above expressions, only the odd terms of *m* are considered due to problem symmetry. The series is then truncated to a finite series and the *α*_*m*_ coefficients are obtained by solving the set of simultaneous linear equations obtained from satisfying Eq 23 at a convenient number of wing stations equal to the number of terms in the series. Finally, the lift and induced drag forces can be obtained from:

$$L=2\int_{0}^{R}\rho V(r)\Gamma (r)dr,$$ (25)

$$D_{i}=2\int_{0}^{R}\rho w_{ef}(r)\Gamma (r)dr.$$ (26)

Thus, the lift and induced drag coefficients are obtained as:

$$C_{L}=\frac{2L}{\rho V_{tip}^{2}{\hat{r}}_{2}^{2}(2R\bar{c})},$$ (27)

$$C_{D_{i}}=\frac{2D_{i}}{\rho V_{tip}^{2}{\hat{r}}_{2}^{2}(2R\bar{c})}.$$ (28)

Note that in the above equations, the lift and induced drag forces are non-dimensionalised using the dynamic pressure at the wing radius of the second moment of wing area.

## Results and Discussion

### Comparison with revolving wing experimental measurements

The revolving wing experiment is a well-known measurement technique employed for insect wing aerodynamic characterisation [31,35–40]. The wing is rotated in the fashion of a simple propeller blade to simulate a continuous down (or up) stroke that excludes the effects that occur at stroke reversal such as wing flipping and wing-wing interactions [37]. In this section, the developed LLT_hw_ is compared to available experimental measurements from revolving wing experiments. Because there are no measurements available for induced drag, only lift coefficient data will be compared here. Although revolving wing experiments have been conducted for different species, the wings used in each case are in close morphological similarity. Thus, the available data does not allow a full validation of the LLT_hw_ against a wide range of planforms. In what follows we compare the LLT_hw_ to three sets of available experimental data for an insect, a bird and a hummingbird.

**Fig 3:**
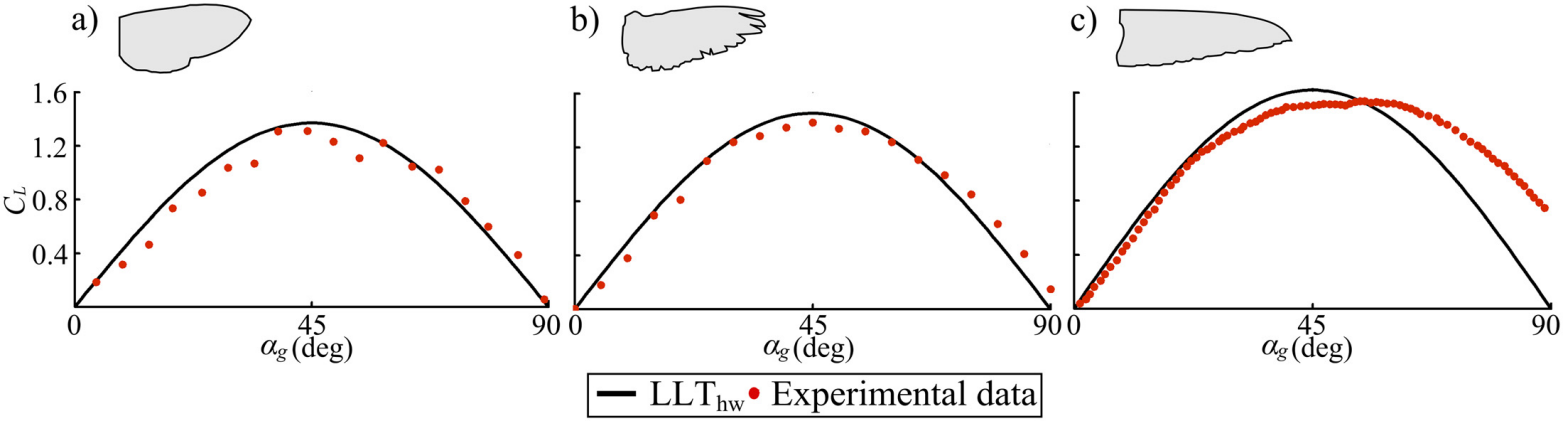
Comparison of lift results with revolving wing experimental measurements. Lift coefficient variation with geometric angle of attack; results evaluated using the LLT_hw_ are compared to available experimental measurements for (a) hawkmoth, experimental data digitised from Fig 6B of [35], (b) pigeon, experimental data digitised from Fig 3A of [37] and (c) hummingbird, experimental data digitised from Fig 6A of [39]; for this case, experimental data beyond 45° are affected by the wing compliance [39].

Usherwood and Ellington [35] provided steady lift coefficient measurements against the geometric angle of attack using a hawkmoth model wing. Later, Usherwood [37] provided similar measurements for pigeon wings at higher Reynolds number. Recently, Kruyt *et al*. [39] provided measurements for hummingbird wings. Note that the hummingbird case is based on measuring a real wing; thus, wing compliance is not fully controlled especially at very high angles of attack [39] and thus the geometric angle of attack has significant uncertainty. Nevertheless, this test case remains useful for comparison against the developed LLT_hw_ especially in the normal operation range of angles of attack (i.e *α*_*g*_ < 45°). The morphological parameters of these three wings are provided in Table 1; these parameters were used as inputs within the LLT_hw_ to calculate the lift coefficient variation with the geometric angle of attack and results are compared in Fig 3. Within the calculation of the three cases, the value of *k*_*per*_ was set to 1.1 whereas by definition the *k*_*flap*_ for a revolving wing is unity.

**Table 1:** Morphological parameters of revolving wings.

Wing	*AR*	${\hat{r}}_{2}$	${\hat{r}}_{1}$(Eq 18)
**Hawkmoth** [35]	2.83	0.511	0.44
**Pigeon** [37]	3.21	0.512	0.443
**Hummingbird** [39]	4.06	0.499	0.43

The results shown in Fig 3 show a good agreement with the experimental measurements in both form and amplitude for the three cases considered. Note that the shape of variation of the lift coefficient with angle of attack is a consequence of the proposed definition of the equivalent angle of attack (see Fig 1b). Of more relevance is the good agreement in the amplitudes of the lift coefficient over the whole first quadrant of angle of attack.

**Fig 4:**
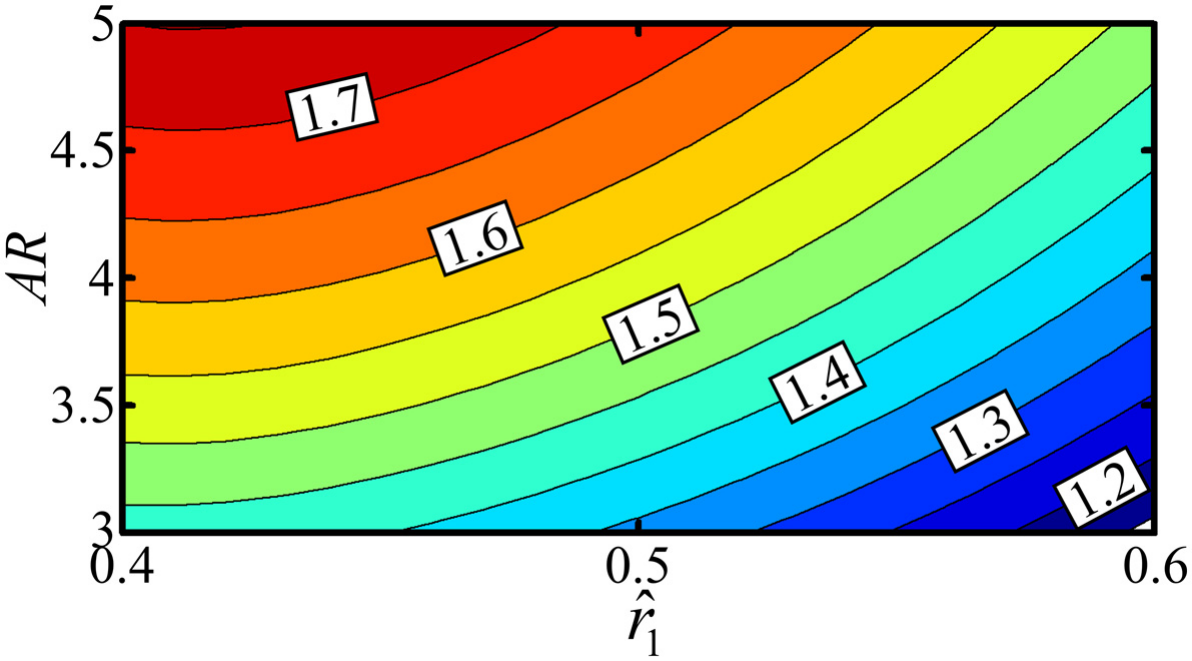
Revolving wing maximum lift coefficient variation. Contours of lift coefficient amplitude against wing aspect ratio and wing area centroid location. The range of values for the aspect ratio and area centroid location were chosen to represent realistic limits for insect wings. In this illustration the value of *k*_*per*_ is set to 1.1 whereas *k*_*flap*_ is unity.

Now the LLT_hw_ is used to evaluate the maximum lift coefficient amplitude (*C*_*L*_ at *α*_*g*_ = 45°) for revolving wings within a range of aspect ratios and chord distributions similar to real insect wings, Fig 4. As expected, the value of the maximum lift coefficient increases as the aspect ratio increases; however, this result must be considered with some caution as an increase in the wing aspect ratio also reduces the chord with respect to the LEV size [55]. Thus, the lift coefficient amplitude increase will stop at some critical point (whose prediction is beyond the capability of the current model) when the LEV size to chord ratio approaches the vortex attachment limit and abrupt stall occurs. Another important result from Fig 4 is that the maximum lift coefficient value decreases as the wing area centroid is shifted towards the tip, despite the fact that having more area towards the tip produces a greater lift force, everything else being equal. Thus, whilst a higher lift force is achieved by having more area towards the tip, a higher lift coefficient is achieved in hovering flight by having more wing area towards the root.

### Application of the LLT_hw_ to insect wings in symmetric normal hovering flight

**Fig 5:**
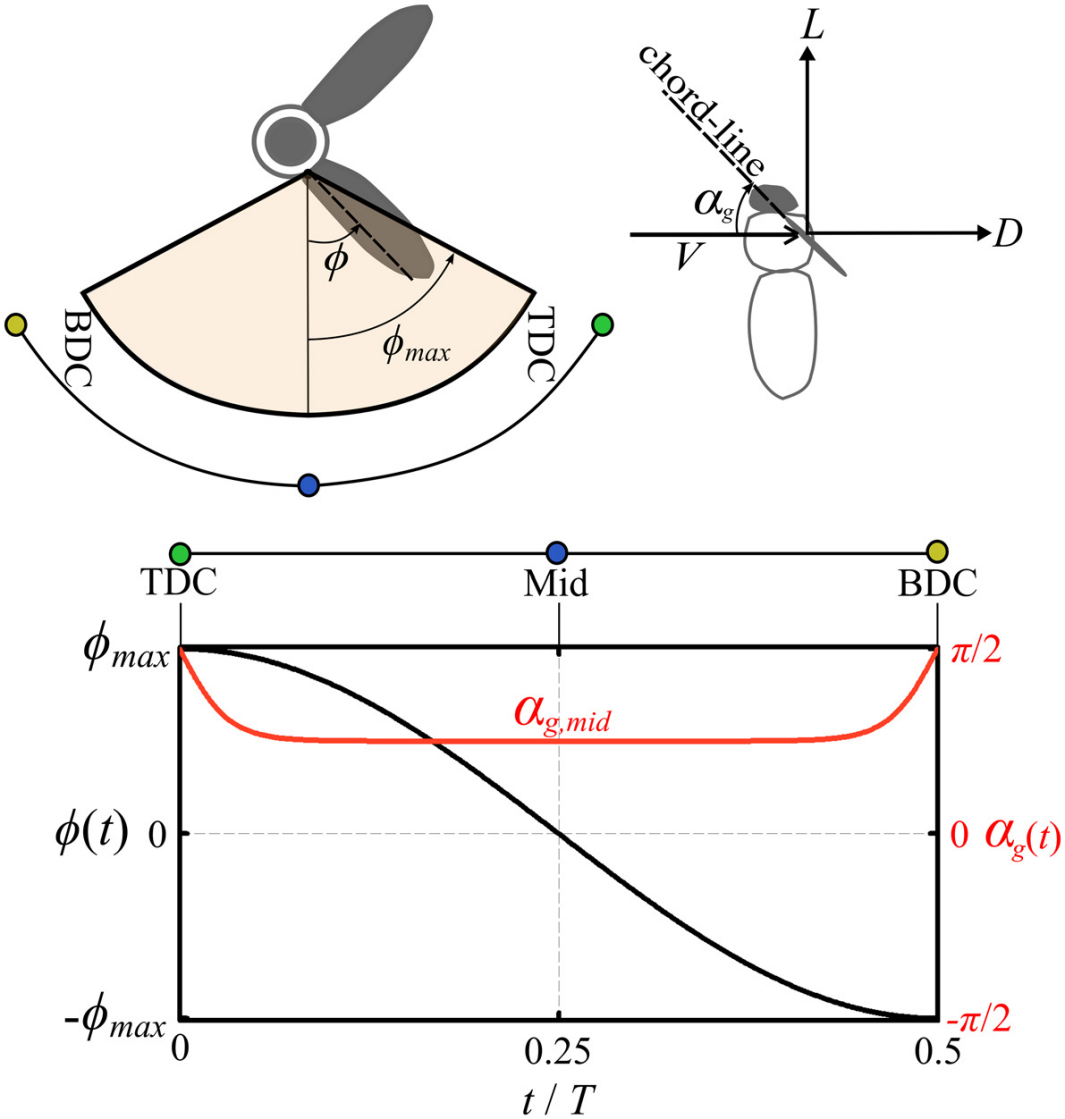
Flapping angle and angle of attack variations in time. Kinematics variation similar to those employed by Sun and Du CFD simulations. Owing to the symmetry of half-strokes, only the down-stroke period is shown. TDC is the cycle top dead centre, BDC is the cycle bottom dead centre and Mid denotes the mid half-stroke.

In this section, the LLT_hw_ is verified against the computational fluid dynamics (CFD) results from Sun and Du [56] that provide comprehensive simulations for a variety of wing shapes operating at different conditions. In their simulations Sun and Du used a horizontal stroke plane, symmetrical half-strokes and a sinusoidal-like variation of flapping angle, Fig 5. The geometric angle of attack was prescribed such that it takes a constant value, *α*_*g*,*mid*_, along a half-stroke and then performs a smooth variation around stoke reversal similar to that shown in Fig 5. Because the flapping cycle half-strokes are symmetrical, only the variation within the down-stroke is shown. Note that the symmetry of the half-strokes also implies that the net mean forces due to rotational and added mass effects are zero [24,29,40,41,57], and only forces due to wing translation contribute to the net mean force production. Table 2 shows the mass, wing geometrical data and motion kinematic data of eight hovering insects that were collected by Sun and Du [56] from the most relevant study of each insect.

**Table 2:** Simulation input data: mass, wing geometry and kinematic parameters for eight hovering insects. Insects ordered by increasing angle of attack.

Insect	mass (mg)	*R* (mm)	$\bar{c}$ (mm)	*${\hat{r}}_{1}$*	*f* (Hz)	*ϕ*_max_ (deg)
**Honey bee (HB)**	101.9	9.8	3.08	0.5	197	65.5
**Dronefly (DF)**	68.4	11.4	3.19	0.48	157	54.5
**Bumble bee (BB)**	175	13.2	4.02	0.49	155	58
**Hoverfly (HF)**	27.3	9.3	2.2	0.52	160	45
**Cranefly (CF)**	11.4	12.7	2.38	0.56	45.5	61.5
**Hawkmoth (HM)**	1648	51.9	18.26	0.46	26.3	60.5
**Ladybird (LB)**	34.4	11.2	3.23	0.47	54	88.5
**Fruit fly (FF)**	0.72	2.02	0.67	0.55	254	75

The main output of Sun and Du simulations [56] are the calculated values of the mid half-stroke geometric angle of attack, *α*_*g*,*mid*_ that would provide weight support (supplied in Table 3). Here we use their *α*_*g*,*mid*_ values to calculate the mean lift force. Table 4 presents the mean lift to weight ratio obtained from the current lifting line theory for the different levels of adaptation employed. The purpose of showing the results for different adaptations is to demonstrate the transparency of the current framework and provide a deeper insight into how these adaptations affect the solution, thus providing more fundamental understanding of the physics of the problem. For example, results are most sensitive to the inclusion of *E* for wings of lower aspect ratios such as for the hawkmoth case. Also, the *k*_*flap*_ value becomes a significant effect when the flapping stroke angle is relatively low as in the hoverfly case; whereas for a case such as the ladybird where the wings scan all the possible area, this effect is negligible. However, the most significant adaption is the inclusion of the non-linear lift curve, which accounts for more than half of the overestimation in the average mean lift to weight ratio. This correction is most significant for the ladybird and the fruit fly, which have higher operating *α*_*g*,*mid*_ values and thus non-linear effects are more pronounced.

**Table 3:** Summary of the main aerodynamic results from Sun and Du CFD simulations.

Insect	HB	DF	BB	HF	CF	HM	LB	FF
*α*_*g*_,_*mid*_ (deg)	25	26	28	29	30	32	43	44
$\bar{L}/W$	1	1	1	1	1	1	1	1
$\bar{P}/mass$ (W.kg^-1^)	41	32	42	27	16	33	28	30

**Table 4:** Mean lift to weight ratio calculated from the LLT_hw_ for different adaptations.

Insect	Linear aero	Nonlinear aero	Nonlinear aero	Nonlinear aero	Nonlinear aero
	*k*_*per*_ excluded	*k*_*per*_ excluded	*k*_*per*_ included	*k*_*per*_ included	*k*_*per*_ included
	*k*_*flap*_ excluded	*k*_*flap*_ excluded	*k*_*flap*_ excluded	*k*_*flap*_ included	*k*_*flap*_ included
	*E* excluded	*E* excluded	*E* excluded	*E* excluded	*E* included
**HB**	1.53	1.34	1.29	1.20	1.11
**DF**	1.72	1.48	1.43	1.29	1.20
**BB**	1.54	1.30	1.25	1.14	1.05
**HF**	1.44	1.20	1.16	1.01	0.96
**CF**	1.71	1.40	1.36	1.27	1.22
**HM**	1.44	1.16	1.11	1.02	0.92
**LB**	1.64	1.09	1.05	1.04	0.96
**FF**	1.72	1.07	1.02	0.98	0.90
**Average ± s.d.**	1.6 ± 0.12	1.26 ± 0.15	1.21 ± 0.15	1.12 ± 0.12	1.04 ± 0.12

**Fig 6:**
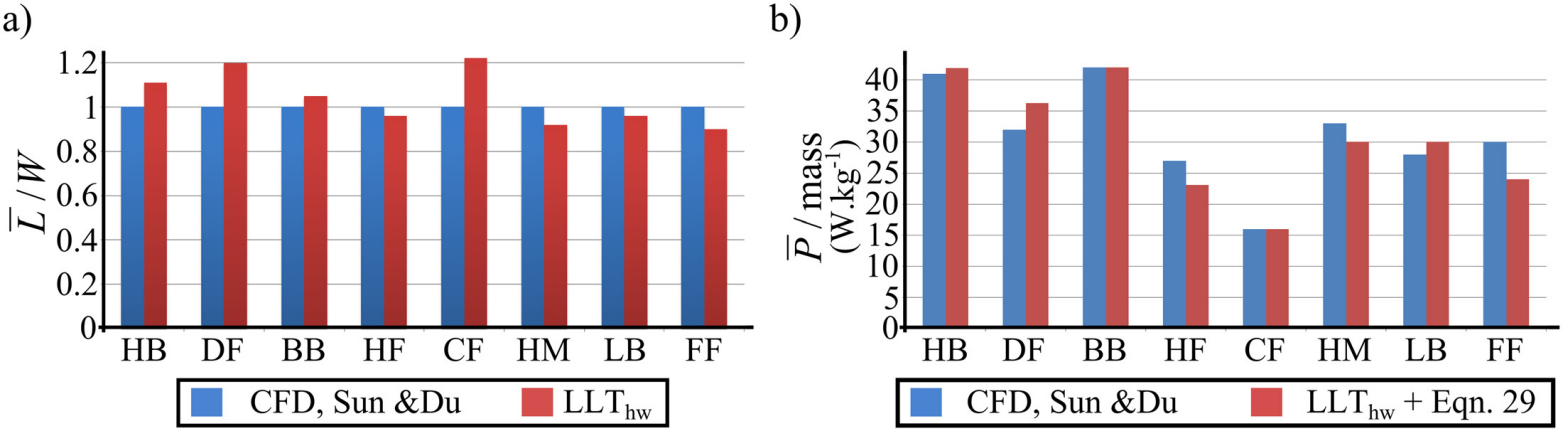
Lift and aerodynamic power evaluation. Comparison of the aerodynamic performance of eight hovering insects from the developed LLT_hw_ against CFD results from Sun & Du; (a) lift to weight ratio and (b) specific aerodynamic power.

It can be seen from the results shown in Table 4 that without including any of the adaptations, the original LLT will always overestimate the lift produced with an average error of +60% for the eight insects. On the other hand, by applying the proposed adaptations, the developed LLT_hw_ formulation is able to predict the mean lift to weight ratio with an average error of 4% compared to the higher order CFD simulations, Fig 6a.

The mean lift to weight ratios obtained from the current model confirm that it is not necessary to account for unsteadiness due to the Wagner effect (indeed, most insect wing aerodynamics predictive models have neglected the Wagner effect, but see models of [57–59]). Sane [34] has tackled this point comprehensively, and using the available experimental evidence he has discussed the lack of influence of the Wagner effect compared to other unsteady effects such as rotational and added mass effects. Nevertheless, Taha *et al*. [57] showed that incorporating unsteady treatments allows better capture of the force time history near stroke reversals. Thus including an unsteady treatment for the quasi-steady models of rotational effects may improve their predictive capabilities for asymmetrical half-strokes where rotational effects can be used for control and manoeuvrability [35,57].

The total drag comprises the induced drag and profile drag containing the effects of skin friction and pressure drag of the wings. Experiments on insect-like wings in simulated hovering flight showed that the skin friction component is negligible, especially at the relatively higher Reynolds numbers (*O*(10^3^) or higher) [31,35–37]. This is consistent with the prevalence of pressure forces over viscous forces at high angles of attack where most insects operate [34]. Thus, with the assumption of zero tangential friction forces, the total drag coefficient can be estimated for an infinitesimally thin flat plat wing using [29,35,38]:

$$C_{D}=C_{L}\text{tan}\alpha_{g},$$ (29)

where *C*_*L*_ is the wing lift coefficient which can be obtained from the developed LLT_hw_. Note that Eq 29 is based on the ‘*normal resultant force*’ model and for more details the reader is referred to any of references [29], [35] or [40]. Despite its simplicity, Eq 29 has been shown to provide effective estimates for the total drag for insect-like flight [25,29,35,40]. The specific aerodynamic power expenditure for the eight insects based on Eq 29 is shown in Fig 6b, and a good agreement is observed compared to the CFD results. This validates the appropriateness of Eq 29 as a model for the total drag.

Predictions of the induced drag can be made directly from the developed LLT_hw_, and Table 5 provides explicit analytical results of the ratio of the induced drag to the total drag. Both the mid half-stroke value as well as the mean flapping cycle value are provided for the induced to total drag ratio in Table 5. In this demonstration we find it more convenient to calculate the mid half-stroke geometric angle of attack, *α*_*g*,*mid*_ that would provide weight support based on the developed LLT_hw_. These *α*_*g*,*mid*_ values are then used to evaluate the aerodynamic quantities in Table 5. Note that for the cases of the fruit fly and the ladybird operation at 45° geometric angle of attack leads to a slightly sub-unity value of the weight support ratio for the given kinematics and wing morphology parameters in Table 2. However, an alteration of the *ϕ*_max_ value by a few degrees can correct for this if required.

**Table 5:** Aerodynamic characteristics calculated from the current LLT_hw_. Insects re-ordered by increasing angle of attack obtained from the LLT_hw_.

Insect	*α*_*g*,*mid*_ (deg)	$\bar{L}/W$	${C_{L}|}_{\alpha_{g,mid}}$	${C_{D_{i}}|}_{\alpha_{g,mid}}$	*${C_{D}|}_{\alpha_{g,mid}}$*	${\frac{C_{L}}{C_{D}}|}_{\alpha_{g,mid}}$	${\frac{C_{L}^{3/2}}{C_{D}}|}_{\alpha_{g,mid}}$	*${\frac{C_{D_{i}}}{C_{D}}|}_{\alpha_{g,mid}}$*	$\frac{{\bar{C}}_{D_{i}}}{{\bar{C}}_{D}}$
**DF**	20.5	1	0.89	0.12	0.33	2.67	2.52	0.35	0.24
**HB**	22	1	0.90	0.13	0.36	2.48	2.35	0.36	0.25
**CF**	22.5	1	1.07	0.14	0.44	2.41	2.50	0.31	0.22
**BB**	26	1	1.02	0.17	0.50	2.04	2.06	0.34	0.25
**HF**	31	1	1.19	0.22	0.72	1.66	1.82	0.31	0.24
**HM**	38	1	1.22	0.25	0.95	1.28	1.41	0.26	0.21
**LB**	45	0.961	1.47	0.26	1.47	1.00	1.21	0.17	0.14
**FF**	45	0.902	1.20	0.27	1.20	1.00	1.10	0.22	0.185

On average for the eight insects, the induced drag is shown to contribute 22% of the total drag based on the mean cycle values and 29% of the total drag based on the mid half-stroke values (Note that the mean cycle values are directly related to the assumed motion kinematic profiles, Fig 5). For insects operating with high mid half-stroke angles of attack (such as the ladybird and the fruit fly) this ratio decreases below 20% for the mean cycle values which is consistent with the expected prevalence of profile drag as the angle of attack increases.

**Fig 7:**
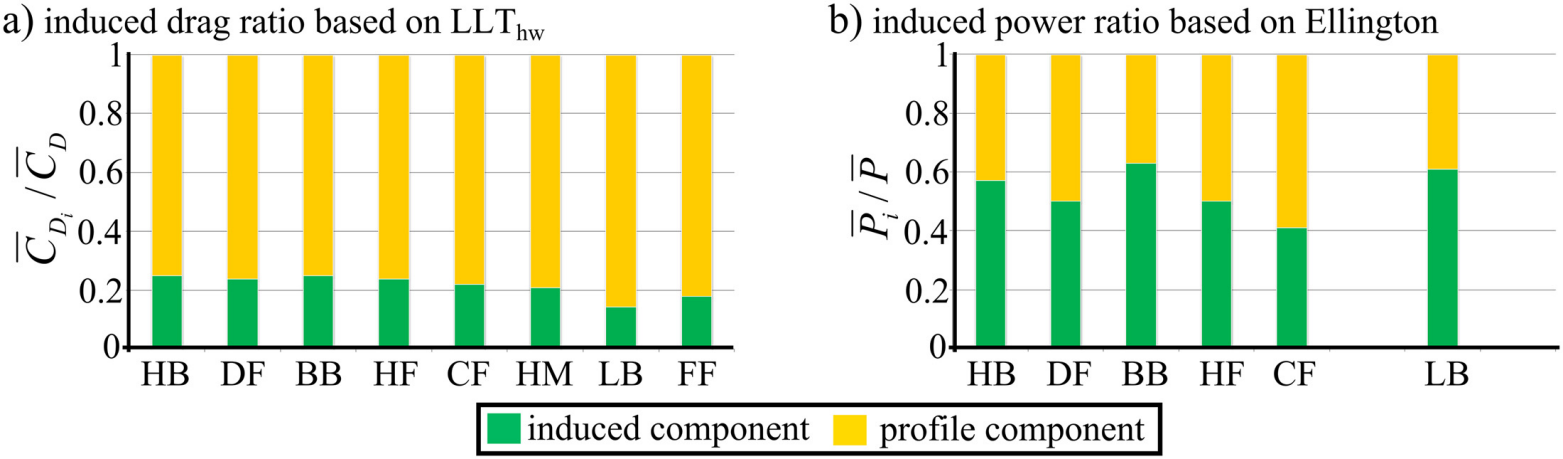
The induced drag contribution in insects hovering flight. Demonstration of (a) the ratio of the induced drag to total drag based on the LLT_hw_, and (b) the ratio of the induced power to the total aerodynamic power based on Ellington's calculations.

The results in Table 5 show that the ratio of induced to profile component is overestimated when employing Ellington's approach [45], Fig 7. Ellington analysed some hovering insects including the ladybird, cranefly, hoverfly, dronefly, honey bee and bumble bee based on measured kinematics and low order methods for evaluating the aerodynamic power, Fig 7b. Based on an average for the considered insects, the ratio of the induced power to the total aerodynamic power was around 0.5. However, the induced power prediction in [45] was based on the Rankine-Froude estimate multiplied by the induced power factor value which had an average value around 1.15 for the considered insects, a value that was shown to be underestimating the induced power factor of normal hovering flyers [30]. Furthermore, the profile power was evaluated based on a low order expression of the profile drag coefficient,$C_{D_{pro}}=7{\text{Re}}^{-1/2}$, an expression based on flow past a cylinder [60]. Whilst this expression was shown to be working at low angles of attack, it becomes unreasonable at high angles of attack.

The obtained drag results confirm that for better aerodynamic efficiency, wings should operate at lower geometric angles of attack. This can be confirmed from the two important aerodynamic performance indices: glide number, *C*_*L*_ */ C*_*D*_ and the power factor $C_{L}^{3/2}/C_{D}$[31] whose values generally decrease within the insects operation range as the mid half-stroke geometric angle of attack increases. Whilst these indices are affected by other parameters and/or variables such as those defining the wing morphology and kinematics, it is clear that the geometric angle of attack value is the parameter that has the greatest influence. The values obtained for these indices are consistent with those obtained from experimental measurements of insect-like hovering wings [31], which are very low compared to fixed wing figures mainly due to the much higher drag associated with the flapping mode of flight.

## Conclusions

A novel lifting line formulation, LLT_hw_, has been proposed for the quasi-steady aerodynamic evaluation of insect-like wings in hovering flight. The developed modelling capability provides a framework to adapt the original LLT for hovering flight and opens the door for simplified yet accurate modelling of 3d lifting surfaces at different operating conditions. The fully theoretical framework allows accurate estimation of the aerodynamics of insect-like wings from geometry and kinematic information alone, as well as providing deeper understanding of the associated aerodynamics in terms of the induced and profile drag associated with the lift production.

The main adaptation proposed is the introduction of the concept of the equivalent angle of attack, which enables the linear aerodynamic LLT formulation to capture the steady non-linear aerodynamics of wings at high angles of attack using a simple analytical correction term. Additionally, a simplified methodology to include a number of non-ideal induced effects within the lifting line theory has been presented. These non-ideal effects are necessary to correctly represent the flapping wing physics, including wake periodicity effects due to discreteness in the wake as well as the effective actuator disk area effect. Finally, low Reynolds number effects as well as the well-known edge velocity correction that improves the LLT performance for various wing planform shapes have been incorporated within the 2d lift curve slope value.

The developed LLT_hw_ has been validated against available measurements from revolving wing experiments for hawkmoth, pigeon and hummingbird wings, and shows very good agreement with respect to both the shape of variation of the lift coefficient with incidence as well as the magnitude. Comparison of the results obtained from the LLT_hw_ and higher order CFD simulations shows that the developed methodology can be judged as a powerful predictive tool for the preliminary evaluation of insect wing aerodynamic performance. The mean lift to weight ratio results have an average error of 4% compared to available CFD results for eight insect cases. The developed model has been used to assess the relative impact of the proposed adaptations on the LLT for the investigated insects. Excluding these adaptations leads on average to a 60% over estimation in the mean lift force required for weight support, and that most of this discrepancy is due to the non-linear lift curve effect. The developed model also provides explicit evaluation of the induced drag component of insect wings. It is shown that on average for the eight insects considered, the induced drag contributes 22% of the total drag based on the mean cycle values and 29% of the total drag based on the mid half-stroke values.

## References

[1] Prandtl L. Tragflügel Theorie. Nachrichten von der Gesellschaft der Wisseschaften zu Göttingen, Ges-Chäeftliche Mitteilungen, Klasse. 1918; 451–477.

[2] Prandtl L. Applications of modern hydrodynamics to aeronautics. NACA TR-116; 1923.

[3] Tietjens OG. Applied hydro- and aeromechanics: based on lectures of L. Prandtl. New York: Dover Publications, Inc; 1957.

[4] Schlichting H, Trunckenbrodt E. Aerodynamics of the airplane. New York: McGraw-Hill, Inc; 1979.

[5] Nabawy MRA, ElNomrossy MM, Abdelrahman MM, ElBayoumi GM. Aerodynamic shape optimisation, wind tunnel measurements and CFD analysis of a MAV wing. Aeronaut J. 2012; 116: 685–708.

[6] Phillips WF, Fugal SR, Spall RE. Minimizing induced drag with wing twist, computational-fluid-dynamics validation. J Aircr. 2006; 43: 437–444.

[7] Rasmussen ML, Smith DE. Lifting-line theory for arbitrarily shaped wings. J Aircr. 1999; 36: 340–348.

[8] Glauert H. The elements of aerofoil & airscrew theory. 2nd ed Cambridge: Cambridge University Press; 1947.

[9] Phillips WF, Snyder DO. Modern adaptation of Prandtl's classic lifting-line theory. J. Aircr. 2000; 37: 662–670.

[10] Jones RT. Correction of the lifting line theory for the effect of the chord. NACA TN-817; 1941.

[11] Sclavounos PD. An unsteady lifting-line theory. J Eng Math. 1987; 21: 201–226.

[12] Mehrle AH. Extension of Multhopp’s quadrature method to cyclic periodic lifting systems. Acta Mech. 2009; 202: 135–144. (10.1007/s00707-008-0017-7)

[13] Anderson JD, Corda S, Van Wie DM. Numerical lifting line theory applied to drooped leading-edge wings below and above stall. J. Aircr. 1980; 17: 898–904.

[14] Anderson JD. Fundamentals of aerodynamics. 5th ed New York: McGraw-Hill; 2011.

[15] Conlisk AT. Modern helicopter rotor aerodynamics. Prog Aerosp Sci. 2001; 37: 419–476. (10.1016/S0376-0421(01)00011-2)

[16] Leishman JG. Principles of helicopter aerodynamics. 2nd ed Cambridge: Cambridge University Press; 2006.

[17] Johnson W. Helicopter theory. New York: Dover Publications; Inc; 1994.

[18] Rayner JMV. Mathematical modelling of the avian flight power curve. Math Meth App Sci. 2001; 24: 1485–1514. (10.1002/mma.196)

[19] Pennycuik CJ. Modelling the flying bird. Oxford: Elsevier; 2008.

[20] Phlips PJ, East RA, Pratt NH. An unsteady lifting line theory of flapping wings with application to the forward flight of birds. J Fluid Mech. 1981; 112: 97–125.

[21] Smith M, Wilkin P, Williams M. The advantages of an unsteady panel method in modelling the aerodynamic forces on rigid flapping wings. J Exp Biol. 1996; 199: 1073–1083. 9318891 10.1242/jeb.199.5.1073

[22] Sane SP. Induced air flow in flying insects. I. A theoretical model of the induced flow. J Exp Biol. 2006; 209: 32–42. (10.1242/jeb.01957) 16354776

[23] Ansari SA, Zbikowski R, Knowles K. Aerodynamic modelling of insect-like flapping flight for Micro Air Vehicles. Prog Aerosp Sci. 2006; 42: 129–172. (10.1016/j.paerosci.2006.07.001)

[24] Sane SP, Dickinson MH. The aerodynamic effects of wing rotation and a revised quasi-steady model of flapping flight. J Exp Biol. 2002; 205: 1087–1096. 11919268 10.1242/jeb.205.8.1087

[25] Berman GJ, Wang ZJ. Energy-minimizing kinematics in hovering insect flight. J Fluid Mech. 2007; 582: 153–168. (10.1017/S0022112007006209)

[26] Whitney JP, Wood RJ. Aeromechanics of passive rotation in flapping flight. J Fluid Mech. 2010; 660: 197–220. (10.1017/S002211201000265X)

[27] Nabawy MRA, Crowther WJ. Aero-optimum hovering kinematics. Bioinspir Biomim. 2015; in press. 10.1088/1748-3190/10/4/044002 26248884

[28] Ellington CP. The aerodynamics of hovering insect flight. I. The quasi-steady analysis. Philos Trans R Soc Lond B Biol Sci. 1984; 305: 1–15. (10.1098/rstb.1984.0049)

[29] Nabawy MRA, Crowther WJ. On the quasi-steady aerodynamics of normal hovering flight part II: model implementation and evaluation. J R Soc Interface. 2014; 11: 20131197 (10.1098/rsif.2013.1197) 24554578 PMC3973364

[30] Nabawy MRA, Crowther WJ. On the quasi-steady aerodynamics of normal hovering flight part I: the induced power factor. J R Soc Interface. 2014; 11: 20131196 (10.1098/rsif.2013.1196) 24522785 PMC3928949

[31] Lentink D, Dickinson MH. Rotational accelerations stabilizes leading edge vortices on revolving fly wings. J Exp Biol. 2009; 212: 2705–2719. 10.1242/jeb.022269 19648415

[32] Ramamurti R, Sandberg WC. A three-dimensional computational study of the aerodynamic mechanisms of insect flight. J Exp Biol. 2002; 205: 1507–1518. 11976361 10.1242/jeb.205.10.1507

[33] Zhao L, Deng X, Sane SP. Modulation of leading edge vorticity and aerodynamic forces in flexible flapping wings. Bioinsp Biomim. 2011; 6: 036007 (10.1088/1748-3182/6/3/036007) 21852729

[34] Sane SP. The aerodynamics of insect flight. J Exp Biol. 2003; 206: 4191–4208. (10.1242/jeb.00663) 14581590

[35] Usherwood JR, Ellington CP. The aerodynamics of revolving wings: I. Model hawkmoth wings. J Exp Biol. 2002; 205: 1547–1564. 12000800 10.1242/jeb.205.11.1547

[36] Usherwood JR, Ellington CP. The aerodynamics of revolving wings: II. Propeller force coefficients from mayfly to quail. J Exp Biol. 2002; 205: 1565–1576. 12000801 10.1242/jeb.205.11.1565

[37] Usherwood JR. The aerodynamic forces and pressure distribution of a revolving pigeon wing. Exp Fluids. 2009; 46: 991–1003. (10.1007/s00348-008-0596-z) 22736891 PMC3380271

[38] Altshuler DL, Dudley R, Ellington CP. Aerodynamic forces of revolving hummingbird wings and wing models. J Zool (Lond). 2004; 264: 327–332. (10.1017/S0952836904005813)

[39] Kruyt JW, Quicazan-Rubio EM, van Heijst GJF, Altshuler DL, Lentink D. Hummingbird wing efficacy depends on aspect ratio and compares with helicopter rotors. J R Soc Interface. 2014; 11: 20140585 (10.1098/rsif.2014.0585) 25079868 PMC4233735

[40] Dickson WB, Dickinson MH. The effect of advance ratio on the aerodynamics of revolving wings. J Exp Biol. 2004; 207: 4269–4281. (10.1242/jeb.01266) 15531648

[41] Wang ZJ. Dissecting insect flight. Annu Rev Fluid Mech. 2005; 37: 183–210. (10.1146/annurev.fluid.36.050802.121940)

[42] Paul RC, Gopalarathnam A. Iteration schemes for rapid post‐stall aerodynamic prediction of wings using a decambering approach. Int J Numer Meth Fluids. 2014; 76: 199–222. (10.1002/fld.3931)

[43] Nabawy MRA, Crowther WJ. Is flapping flight aerodynamically efficient? 32nd AIAA Applied Aerodynamics Conference, AIAA Aviation and Aeronautics Forum and Exposition, 16–20 June 2014, Atlanta, Georgia. (10.2514/6.2014–2277)

[44] Ellington CP. The aerodynamics of hovering insect flight. V. A vortex theory. Philos Trans R Soc Lond B Biol Sci. 1984; 305: 115–144. (10.1098/rstb.1984.0053)

[45] Ellington CP. The aerodynamics of hovering insect flight. VI. Lift and power requirements. Philos Trans R Soc Lond B Biol Sci. 1984; 305: 145–181. (10.1098/rstb.1984.0054)

[46] Birch JM, Dickson WB, Dickinson MH. Force production and flow structure of the leading edge vortex on flapping wings at high and low Reynolds numbers. J Exp Biol. 2004; 207: 1063–1072. (10.1242/jeb.00848) 14978049

[47] Pope A. Basic wing and airfoil theory. New York: McGraw-Hill, Inc; 1951.

[48] Abbott IH, Von Doenhoff E. Theory of wing sections. 2nd ed New York: Dover Publications, Inc; 1959.

[49] Ellington CP. The aerodynamics of hovering insect flight. II. Morphological parameters. Philos Trans R Soc Lond B Biol Sci. 1984; 305: 17–40. (10.1098/rstb.1984.0050)

[50] Ellington CP. The novel aerodynamics of insect flight: applications to micro-air vehicles. J Exp Biol. 1999; 202: 3439–3448. 10562527 10.1242/jeb.202.23.3439

[51] Traub LW. Analysis and estimation of the lift components of hovering insects. J Aircr. 2004; 41: 284–289.

[52] Okamoto M, Yasuda K, Azuma A. Aerodynamic characteristics of the wings and body of a dragonfly. J Exp Biol. 1996; 199: 281–294. 9317808 10.1242/jeb.199.2.281

[53] Azuma A. The Biokinetics of Flying and Swimming 2nd ed *AIAA Educational Series*, AIAA, Reston, Virginia; 2006.

[54] Grădinaru S. Pairs of Glauert integrals. Analele Universității Spiru Haret—Seria Matematică-Informatică, 2011; 7: 19–24.

[55] Harbig RR, Sheridan J, Thompson MC. The role of advance ratio and aspect ratio in determining leading-edge vortex stability for flapping flight. J Fluid Mech. 2014; 751: 71–105. (10.1017/jfm.2014.262)

[56] Sun M, Du G. Lift and power requirements of hovering insect flight. Acta Mech Sin. 2003; 19: 458–469. (10.1007/BF02484580)

[57] Taha HE, Hajj MR, Beran PS. State-space representation of the unsteady aerodynamics of flapping flight. Aerosp Sci and Technol. 2014; 34: 1–11. (10.1016/j.ast.2014.01.011)

[58] Walker JA, Westneat MW. Mechanical performance of aquatic rowing and flying. Proc R Soc Lond B Biol Sci. 2000; 267: 1875–1881. 10.1098/rspb.2000.1224 PMC1690750 11052539

[59] Walker JA. Rotational lift: something different or more of the same. J Exp Biol. 2002; 205: 3783–3792. 12432002 10.1242/jeb.205.24.3783

[60] Wang ZJ. The role of drag in insect hovering. J Exp Biol. 2004; 207: 4147–4155. 15498960 10.1242/jeb.01239

