# Correction: A Quasi-Steady Lifting Line Theory for Insect-Like Hovering Flight

**DOI:** 10.1371/journal.pone.0158929

**Published:** 2016-07-05

**Authors:** The PLOS ONE Staff

The second author’s name is spelled incorrectly. The correct name is: William J. Crowther. The correct citation is: Nabawy MRA, Crowther WJ (2015) A Quasi-Steady Lifting Line Theory for Insect-Like Hovering Flight. PLoS ONE 10(8): e0134972. doi:10.1371/journal.pone.0134972. The publisher apologizes for the error.
